# Multi-Bolted Connection for Pultruded Glass Fiber Reinforced Polymer’s Structure: A Study on Strengthening by Multiaxial Glass Fiber Sheets

**DOI:** 10.3390/polym14081561

**Published:** 2022-04-11

**Authors:** Quang Duc Tran, Phan Viet Nhut, Yukihiro Matsumoto

**Affiliations:** 1 Department of Architecture and Civil Engineering, Toyohashi University of Technology, Toyohashi 441-8580, Aichi, Japan; phan.viet.nhut.yu@tut.jp (P.V.N.); matsumoto.yukihiro.lp@tut.jp (Y.M.); 2 Department of Civil Engineering, University of Technology and Education-The University of Danang, Da Nang 550000, Vietnam

**Keywords:** pultruded GFRP, glass fiber sheets, failure modes, strengthening, multi-bolted connection

## Abstract

Pultruded Glass Fiber Reinforced Polymers (PGFRPs) are becoming a new mainstream in civil construction because of their advantageous properties. One of two main elements, glass fibers, have been constructed by unidirectional glass roving in applicate progress. PGFRPs do not have high shear strength, which is determined by another element is the matrix. In the future, the demand for enhanced serviceability of existing PGFRP structures could be seen as unavoidable. Therefore, multi-bolted connection being the most typical type of connecting member, strengthening the connection performance of PGFRPs through connection is necessary. Previous researchers have studied several methods for improving connection capacity, including pasting glass fiber sheets (GFS). However, experimental research is lacking for multi-bolted connection. This study investigated several strategies of specimens, including the quantity of bolts (two bolts, four bolts, and five bolts); the end distance/diameter ratio (*e* = *2d*; *e* = *3d*) under tensile load; and three types of glass fiber sheets (GFS) (0°/90°, ±45° and chopped strand mat (CSM)). The experiment’s results showed the strengthening effects and the failure mode on the specimens. These findings could address the gap in knowledge that needs to be resolved with respect to PGFRPs’ composite design, through evaluation and discussion of their behavior.

## 1. Introduction

Pultruded glass fiber reinforced polymers (PGFRPs) have become the most popular FRP material, widely used in industry and construction. The advantageous properties that are the counterpart to changing from conventional materials to PGFPRs consist of light weight, high strength, stiffness, etc. [1,2]. Pultruded techniques were reviewed by Bank [3]. Manufacturers use glass fiber constituents to improve the stiffness and strength of plastics.

Several advanced properties of PGFRPs, such as their resistance to chemicals, their nonmagnetic nature, their isothermal properties, their electrical conductivity, their fatigue resistance, and their easy installation, make PGFRPs an exciting to alternative traditional construction materials [3,4]. One of the largest markets for PGFRPs in the construction field is for pedestrian bridges [5,6]. PGFRPs have shown long durability in various situations when subjected to long-term environmental effects [7], which allows reduction in expenditures on maintenance work. Recently, pultruded GFRP reinforcement was used to manufacture railway sleepers [8] and concrete slabs [9], and in other general applications [10]. Other typical applications of PGFRPs included building structures and elements [11,12] and a marine construction/wastewater treatment plant, overcoming the corrosion problem in a severe sea or chemical environment [13,14]. 

Several the standard for the design of PGFRP materials are “the Pre-Standard for Load & Resistance Factor Design (LRFD) of Pultruded Fiber Reinforced Polymer (FRP) Structures” (ASCE 2010 submitted to the American Composites Manufacturers Association (ACMA)); “Prospect for new guidance in FRP design” in 2016, which reviewed the previous guidebook; “Structural Design of Polymer Composites,” (the EUROCOMP Design Code and Handbook in 1989) [15,16,17].

The application of PGFRPs has convenience and economy, and the bolted connection is the most popular joint connection type for PGFRPs. During the development of PGFRPs’ applications, more issues appeared in the structural design of bolted connections. Some studies investigated the connection problem, and their results have been highlighted [18,19]. To identify aspects of PGFRPs’ bolted connection failure modes, several authors implemented experimental, and some studies by theoretical method [20,21,22,23].

Ascione et al. [24] investigated the effects of fiber direction on bearing failure strength on GFRPs that were pin bearing bolted. Three kinds of laminate were studied, with several values of angle created to form fiber direction and external force. There were sixteen values of angle for type 1 laminate and seven values for types 2 and 3. The result showed a linear decrease in the ultimate load, depending on the bolt diameter. The authors proposed a formula for predicting the ultimate bearing load for directional fiber angles and bolt diameters. Prabhakaran et al. [25] also conducted an experiment to study the pultruded direction effect on multi-load direction. Despite differences in the types of PGFRP (bonded by vacuum and pultruded) and in the off-axis angles (different values), the results of these two studies were similar. 

Other authors have investigated other parameter inputs. Chao Wu et al. [26] and Persson and Eriksson [27] researched static and fatigue performance on steel and blind bolts. Cooper and Turvey [28] investigated clamping force. Wang [29] studied bolt-hole size and clearance aspects.

Based on previous research investigating bolted failure modes, four main modes have been reported: bearing, net-tension, shear-out, and cleavage [17,18,19,20]. Cooper and Turvey in 1995 and Turvey in 1998) [23,28] (parts G and H) showed different modes for connections having multiple rows of bolts (Prabhakaran et al., 1996 [22]; Hassan et al., 1997 [30]; Wang, 2004 [31]).

Bearing failure is preferably due to its progressive failure process [25,26,29]. The other failure modes are brittle and catastrophic [25]. However, experimental results also showed that a pseudo-ductile shear failure became possible by increasing the end distance (Abd-El-Naby and Hollaway 1993a) [32]. Mottram and Turvey (2003) [33] demonstrated that failure modes could be changed by varying the geometric parameters, such as the end distance to bolt diameter ratio and the edge distance to bolt diameter ratio.

The major material issues, such as bolted connection or mechanical properties, were also summarized in several papers [18,34,35]. Some authors have reviewed the recent research and development trend regarding general issues of PGFRPs in civil and structure applications [35,36,37]. 

The joint strength is commonly estimated by the bolt connection in PGFRPs, rather than the profile member. In contrast, the capacity of the connections is determined by the shear or bearing strength of the material. In this study, strengthening by advanced material was investigated as a potential method for increasing the strength of PGFRP connections, in addition to end distance and bolt quantity. Some authors have developed strength of structure by using strengthening layers and pasting them to ordinary materials, sometimes combined with increased bolt number or end distance. Nhut et al. (2021) [38,39] implemented an experiment in strengthening a single bolt connection by using the glass fiber sheet (GFS). The result showed a noticeable increase in the development of connection strength. GFS, which is made from glass fiber and epoxy resin, as explained in Section 3, was considered a cost-effective material for upgrading the strength of PGFRPs by Uddin (2004) [40]. Other authors have investigated other materials, including carbon nanotube, nano clay, or metal inserts, to improve the performance of bolt connections in composite structures [41,42,43,44].

In summary, the review of the research literature shows clearly the advantageous properties and applicability of PGFRPs. Many studies have tried to improve the performance of materials in various aspects, including the important factor of bolted-connection strength. However, there has been no article that has investigated strengthening PGFRPs by GFSs with multi-bolted connection. Therefore, it was necessary to implement testing and evaluation of the effectiveness of the strengthening method by GFSs for bolted-connection structures.

In addition, the parameters of specimens were considered by referring to the previous studies. Some studies concluded that the bearing load of a connection is enormous when the direction load-fiber angle reduces [24,25]. This study is focused on a connection test with a direction load-fiber angle of zero. Moreover, many authors demonstrated that failure modes could be changed by varying the geometric parameters, such as the edge distance to bolt diameter ratio and the edge side distance to bolt diameter ratio. In this study, we tried to apply the GFS as a potential strengthening material in several conditions, including the two most crucial aspects: the number of bolts and the end distance of the connection area. The input parameters of the specimens included GFSs and end distance. Based on the testing results, this article evaluated and proposed an effective method for strengthening by using GFSs for the multi-bolt connection of PGFRP structures.

## 2. Experimental Design

### 2.1. Connection System

A bearing-type connection is one where the transfer of the connection force is entirely by the bearing between the shaft(s) of the bolting and the connecting components [15]. In this study, a 21 N.m torque force was applied when setting up the bolt connections for the specimens (ISO 6789-1:2017). Nevertheless, for the design of bearing-type connections, it was assumed that there is no force transferred through friction between the connected elements in the connection.

### 2.2. Bolts and Bolt Holes

ASCE standards [15] instruct those bolts shall be of carbon or stainless steels with specifications in accordance with ASTM standards A307, A325, or F593. Bolts shall be in the range of diameters, d, from 3/8 of an inch (9.53 mm) up to, and including, 1 inch (25.4 mm). The bolt length shall be such that the end of the bolt extends beyond or is at least flush with the outer face of the nut when properly installed. The length of the bolt shank with thread that is in bearing with FRP material should not exceed one-third of the thickness of the plate component. Bolts shall be torqued to the snug-tightened condition. The slope parts in contact with the washer, the bolt head, and the nut shall be equal to or less than 1:20 with respect to a plane that is perpendicular to the bolt axis.

The nominal hole diameter, *d_n_*, shall be 1/16 of an inch (1.6 mm) larger than the nominal bolt diameter, d. Holes must be drilled or reamed. Oversized holes greater than 1/16 of an inch (1.6 mm) larger than bolt shall not be permitted, and slotted holes shall not be aligned in the primary direction of connection force.

**Figure 1:**
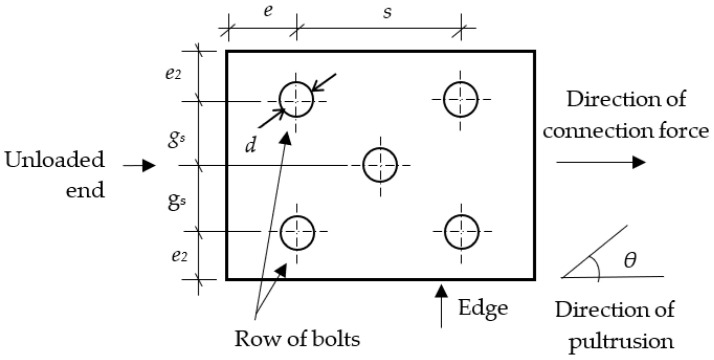
Connection definition.

Bolts, bolt holes, and connection geometries were determined based on the minimum requirements of the ASCE standard [15], as shown in Figure 1 and Table 1. In this study, the bolt is M12 and bolt hole size is 13.5 mm.

### 2.3. Prediction of Modes of Failure

**Figure 2:**
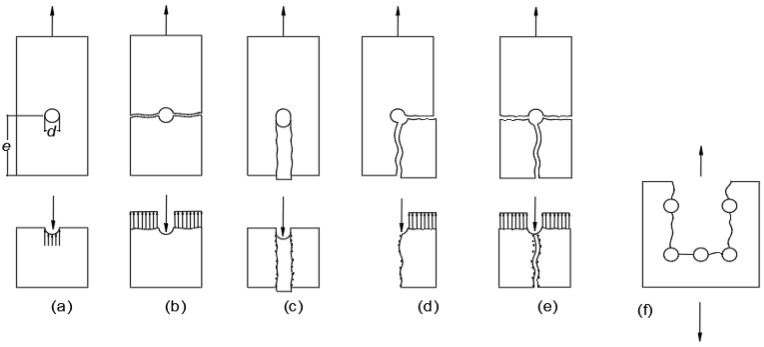
Failure mode of bolted connection and simplified stress distributions. (**a**) bearing, (**b**) net-tension, (**c**) shear-out, (**d**) cleavage, (**e**) net-tension ‘splitting’, and (**f**) block shear.

Figure 2 shows the primary in-plane failure of plate-to-plate connection, with (a) to (e) showing different failure modes of single-bolted connections [23,28] or multi-bolted connection [25,31].

The other failure modes illustrated in Figure 2 are not desirable because their failure mechanisms are sudden. Under most geometrical arrangements it is found that bolted connections with two and three rows of bolts will have faster failure modes, either of net-tension (Hassan et al., 1997) [30] or a form of block shear (Prabhakaran et al., 1996) [45].

## 3. Specimens’ Material

### 3.1. PGFRP Material

A commercial product of the Fukui Fibertech Co., Ltd. (Toyohashi, Aichi, Japan), which is named FS1005, comprises three phases of constituents, continuous direction glass roving (CD), fiber glass fiber mat (GFM), and unsaturated polyester resins, which were used to make specimens. The manufacturer used a special bond to combine those parts into a PGFRP profile sheet.

**Figure 3:**
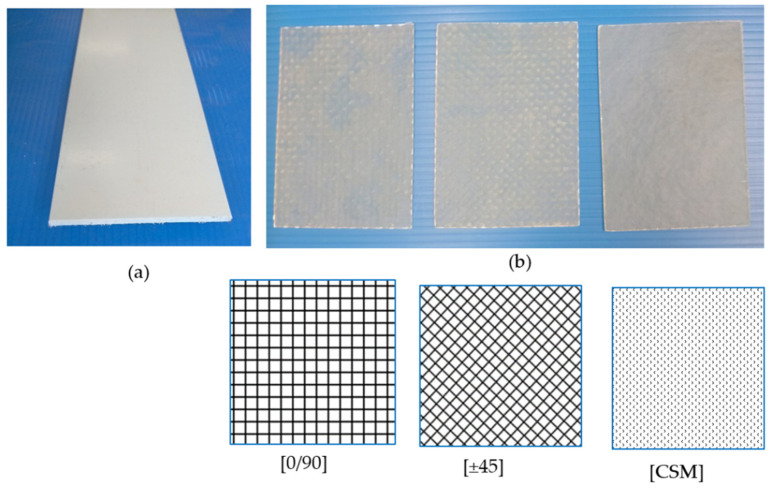
A schematic sectional view of (**a**) PGFRP original material sheet, and (**b**) GFS sheet after mound.

**Figure 4:**
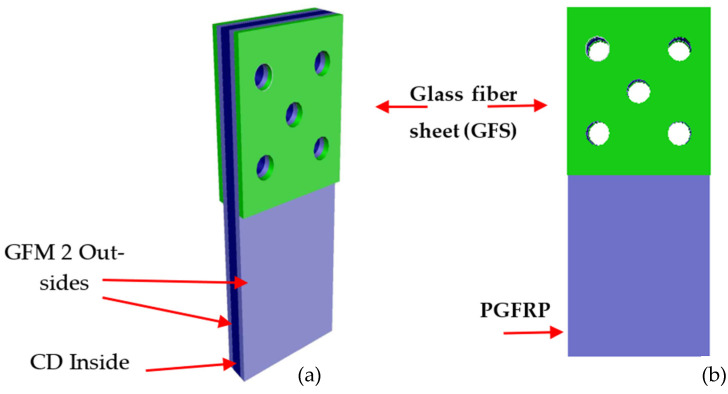
Strengthening diagram of GFSs for PGFRP connections: (**a**) perspective view of PGFRP with element section, (**b**) front view.

The original plate, shown in Figure 3, has an average thickness of 5 mm. The 3D model shown in Figure 4 also describes the detail of a PGFRP, which includes 0.5 mm of the outside GFM part’s thickness and 4 mm thickness of the inside CD part. The dimensions of the specimens were determined to meet minimum criteria that corresponded with bolt diameters and row bolts based on the ACSE pre-standard [15]. The center part of the PGFRP sheet was cut to 84mm in width to make specimens for the tensile test. Then, the GFSs were bonded onto both sides of the PGFRP plate using E250 adhesive (product of Konishi, Osaka, Japan) to finish creating the specimens.

### 3.2. Strengthening by Fiber Sheet

The study used three types of glass fiber sheet (GFS), represented by the green sheet in Figure 4, to investigate the effect and failure models of specimens after strengthening. Two types of original glass fiber sheets used were 0°/90° woven roving (ERW580-554A) and CSM (ECM450-501) (products of the Central Glass Co., Ltd., Tokyo, Japan with weights of 580 (g/m^2^) and 450 (g/m^2^), respectively). From the first type, three layers of 0°/90° were stacked, then cut to [0/90] or rotated onto ±45° to make [±45] lamination. [CSM] was made by a similar method from CSM. These three layers were made adhesive by the VaRTM molded method, as shown in Figure 3. The VaRTM method can reduce the thickness of various layers of fiber content. In a previous study, Nhut (2021) [39] proposed detailed GFSs procedure making.

## 4. Experiment Procedure

### 4.1. Setup and Instrumental for Connection Tests

In this study, a tensile test was conducted to investigate the strength of the bolted connection. Table 2 shows the test program for the PGFRP connection with a list of 24 specimen types, combined from three parameters: quantities of the bolt, material of GFS, and end distance. Each type was included in three samples, which meant a total of 72 samples were used in the test. The thicknesses of the GFSs were measured after molding and before sticking them on the PGFRP surfaces. In the table:

- NS_*T*-*N*_ is the non-strengthened specimen;
- [0/90]_*T*-*N*_, [±45]_*T*-*N*_, and [CSM]_*T*-*N*_ are the specimens strengthened by (on both sides) 0°/90°GFS, ±45° GFS, and CSM GFS;
- *T* is the ratio of end distance (*e*) and bold diameter (*d*) (*e = 2d* and *e = 3d*);
- *N* is the number of bolts, respectively (*N* = 2; 4 and 5).

### 4.2. Expanding the Strengthening Area for the Connection Tests

An additional test for determining failure mode occurred in [±45] and [0/90] GFS specimens when a GFS’s area was extended. The distance from the edge in the loaded end to the nearest row bolts was equivalent to four and five times the bolt-dimension (denoted by *4d* and *5d*). Table 3 provides a list of the details for testing specimens with an expanded GFS area.

**Figure 5:**
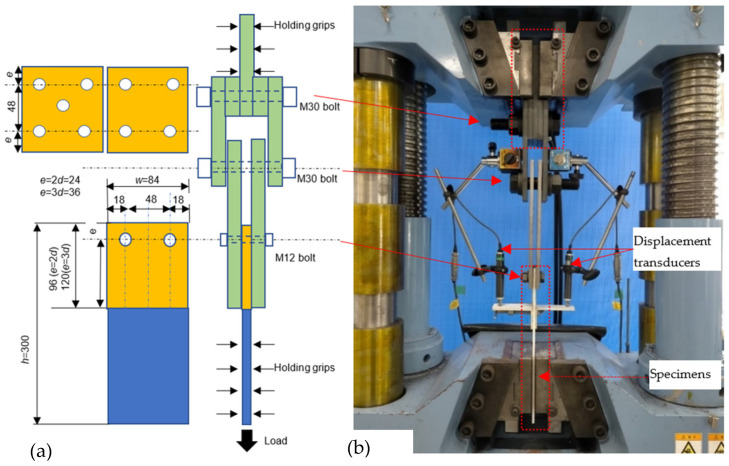
(**a**) Specimen configuration with five full bolts, and (**b**) test setup in tensile tests. Unit: mm.

The experiment used a 1000 kN Maekawa tensile testing machine, as shown in Figure 5.

## 5. Results and Discussions

### 5.1. Failure Modes of the Specimens in the PGFRP Connections

**Figure 6:**
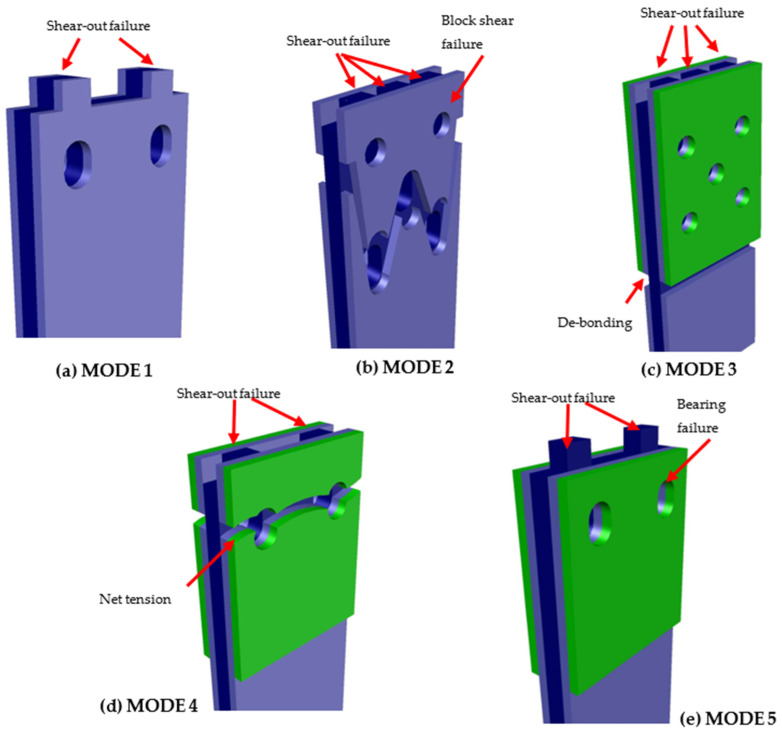
Failure modes of the PGFRP connections.: (**a**) MODE1, (**b**) MODE2, (**c**) MODE3, (**d**) MODE4, (**e**) MODE5.

**Figure 7:**
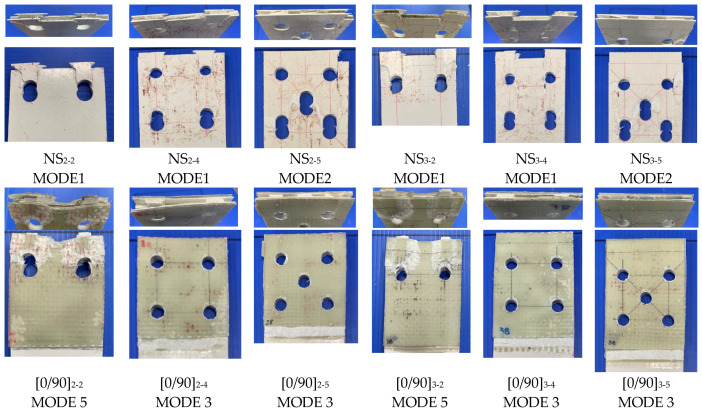

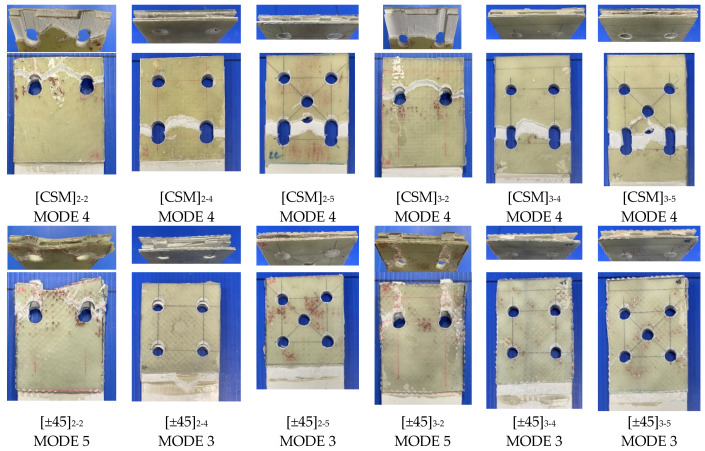
Typical failure modes of the PGFRP connections in the experiment.

Five main types of failure modes occurred in the connection strength experiment. The typical failure modes are simulated as 3D views in Figure 6. Pictures resulting from the experiment are provided in Figure 7 with perspective and front views, which were observed for each typical specimen.

The failure modes were combined from two or three elements’ details, as shown in Table 4.

Before explaining the reason, the definition of failure modes is briefly described, as follows:

- MODE 1 was a shear-out failure in both the GFM and CD layers in two bolts and four bolts with non-strengthened specimens (NS).
- MODE 2 is a two-element failure mode: shear-out inside (CD layer) and block shear failure outside (GFM layer), which occurred in five-bolt NS specimens.
- MODE 3 is a combined failure mode with shear-out in the CD layer as GFM and GFS de-bonded together. This failure mode occurred in four- and five-bolt specimens with [0/90] and [±45] GFS.
- MODE 4 was obtained in all CSM-strengthened specimens (two, four, and five bolts). It consists of net-tension in the GFS and GFM parts and shear-out in the GFM part.
- The MODE 5 failure type was a bearing in the GFS/CD part and shear-out in the CD part. This mode was taken in [0/90] and [±45] GFS specimens with two bolts.

The failure mechanism was evaluated based on the two components of the strengthening specimens, CD on the inside and GFM/GFS combined layer on the outside. The failure tended to happen at the weakest component strength. The ACSE standard [15] proposed measure was used to calculate the nominal strength of the bolted connections with two or three rows of bolts. The nominal connection strength, Rn, was taken as the minimum of *R_bt_*, *Rtt*, *R_br_*, *R_nt_,_f_*, *R_sh_*, and *R_bs_*, where:

- *R_bt_* = Bolt strength;
- *R_tt_* = Tension (through-the-thickness) strength;
- *R_br_* = Pin-bearing strength;
- *R_nt,f_* = Net-tension strength at the first bolt row;
- *R_sh_* = Shear-out strength;
- *R_bs_* = Block shear strength for concentric load; and
- *R_bs,e_* = Block shear strength for eccentric load

Due to the fact that the tensile force in the test was the concentric load, *R_bs,e_* was not considered in the calculation. The debonding failure component occurred in all specimens except in MODE 1. After debonding, failure corresponded with the weakest (minimum) component strength. Using this principle, the failure mode in the experiment can be explained by calculating the component strength of the specimens.

**Figure 8:**
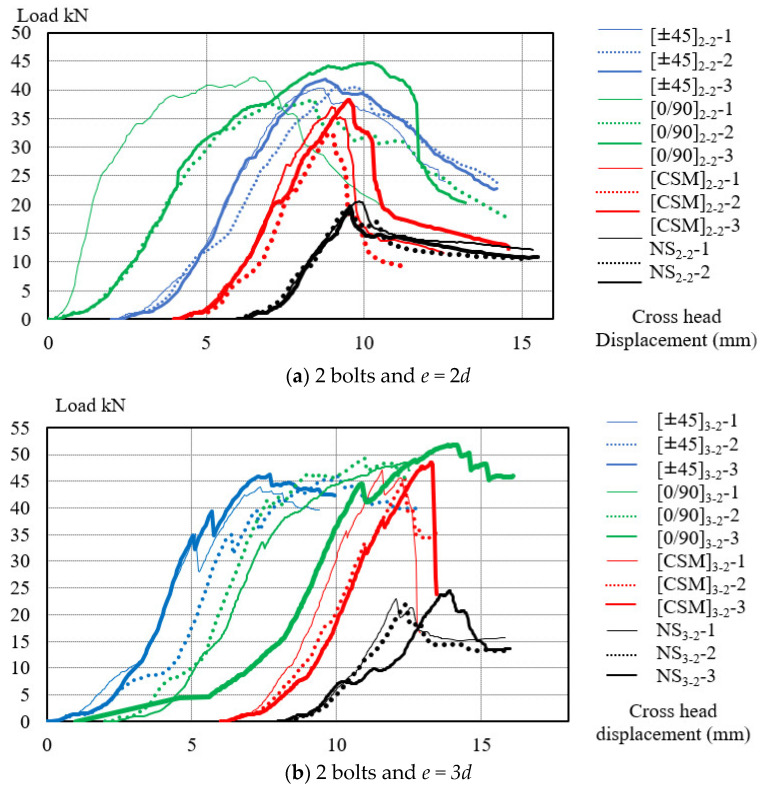

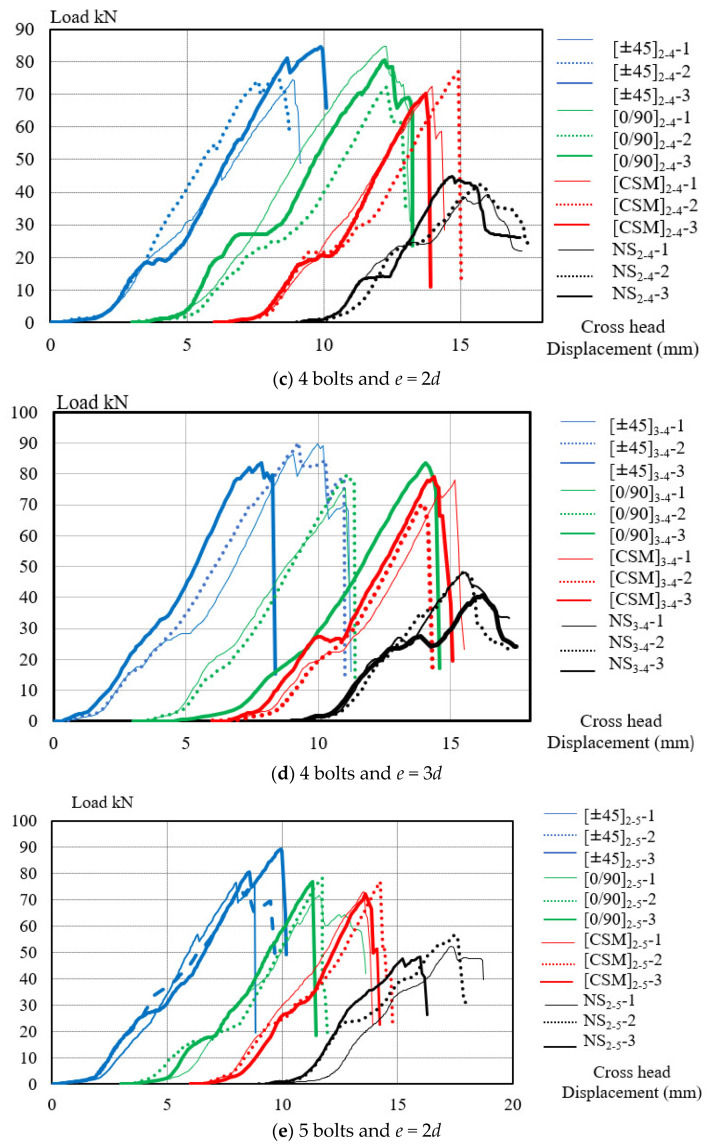

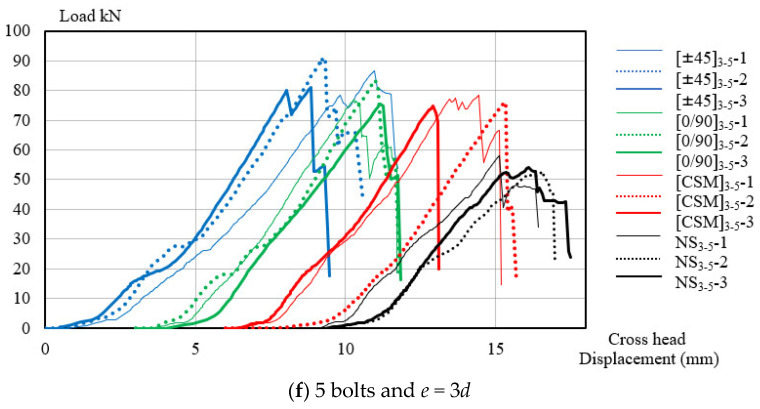
Load-cross head displacement relations in the PGFRP connections of all specimens: (**a**) 2 bolts and *e =* 2*d*; (**b**) 2 bolts and *e =* 3*d*; (**c**) 4 bolts and *e =* 2*d*; (**d**) 4 bolts and *e =* 3*d*; (**e**) 5 bolts and *e =* 2*d*; (**f**) 5 bolts and *e =* 3*d*.

The estimated values of component strength are shown in Appendix A and the results of load-cross head displacement are shown in Figure 8. 

The tendencies of the failure modes are explained as follows:

- MODE 1 occurred in all thicknesses of NS two and four bolts. The results met with previous studies’ results that investigated the failure mode in the base plate PGFRP. The shear-out strength of the CD layer is much less in comparison with the bearing or tensile strength. Therefore, the shear-out failure mode has appearance in CD and lead to GFM layer shear-out meanwhile the loading increases.

From MODE 2 to MODE 5, based on the observation, debonding failure occurred in whole specimens. During the developing of loading, each component failed with the mode, depending on the order of its component strength size, as indicated in Appendix A.

**Figure 9:**
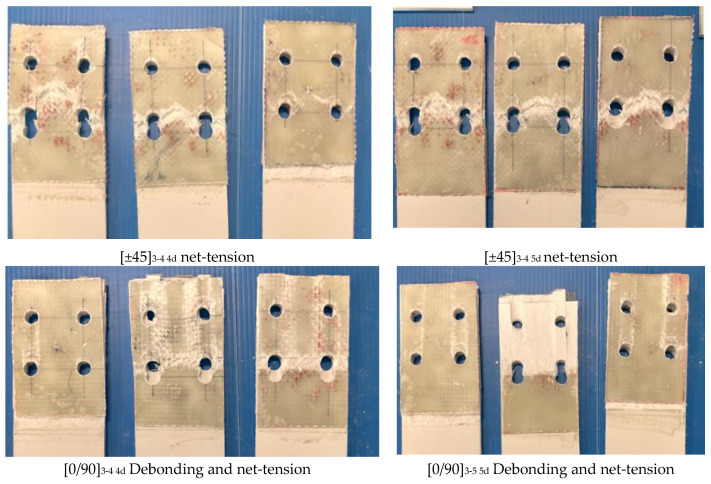
Typical failure modes of the PGFRP connections in the expanded GFS specimens.

- The other mode in NS is MODE 2, the block shear failure mode, which occurred with three-bolt rows in five-bolt specimens. As shown in Appendix A, block shear strength was considered as the weakest. After block shear failure occurred, the second component failure came with shear-out of the inside layer (CD), corresponding with the order of strength size.
- The debonding failure witnessed in MODE 3 occurred in the whole GFS strengthening area. As indicated by the ASCE [15] principle, bonding strength tended to increase to the combined strength of the bearing or shear-out strength of GFM/GFS before debonding. However, due to debonding occurring in the whole surface of the GFM/GFS area, only in the CD layer, which weakest with shear-out strength, was failure consequently.
- By a similar method, MODE 4 failure in the [CSM] specimens can be explained. After loading reached the lowest combined strength (the tensile strength) the net-tension failure occurred. Consequently, the CD layer inside also demonstrated shear-out. In *e = 2d* and two-bolt specimens, the tensile and shear strength in GFM/GFS were equivalent, thereby leading to the “hybrid mode” in which shear-out and net-tension failure co-occurred.
- With reference to Appendix A, the combined bearing strength of GFM/GFS was lower than others. Therefore, MODE 5 occurred in [0/90] and [±45] GFS with two-bolt specimens corresponded with the bearing failure modes.
- There was a distinction in the failure modes of GFS area-expanded specimens. The net-tension occurred in all specimens [±45]_3-4 4d_ and [±45]_3-4 5d_. The debonding was a major failure mode in [0/90]_3-4 4d_ and [0/90]_3-4 5d_, as depicted in Figure 9. Previously, Nhut [46] measured the tensile stress of [±45] and [0/90]. The result was that the tensile stress of the [0/90] specimen was two times higher than that of the [±45] specimen, which is the major reason explaining the difference in the failure modes.

### 5.2. Strengthening Effects of GFSs on the PGFRP Connections

#### 5.2.1. Maximum Load

Figure 8 shows the crosshead loads-displacements relation diagram of all specimens in the PGFRP connections. All types of GFS or non-strengthening specimens were divided into groups in which the specimens had the same parameters of end distance/bolt diameter ratio (e/d) and number of bolts.

There were six groups:

1. Two bolts and *e* = 2*d*;
2. Two bolts and *e* = 3*d*;
3. Four bolts and *e* = 2*d*;
4. Four bolts and *e* = 3*d*;
5. Five bolts and *e* = 2*d*;
6. Five bolts and *e* = 3*d*

The average values of displacement were obtained from the crosshead, as shown in Figure 5. The numbers 1, 2, and 3 at end of the name code represent three samples for each type of specimen. The initial points in the lines were moved and adjusted in the graph to provide a better overall view of all the load-relative displacement relationships.

Figure 8a,b shows the load-displacement relations of two-bolt specimens. After reaching the maximum load, loading in the [0/90] and [±45] GFS specimens with two bolts was maintained for a period before dropping. This is because bearing failure occurred in the GFSs (MODE 5). In the other failure modes, the bearing load rapidly decreased after reaching the ultimate load. The maximum load corresponding to reduction in the point of stiffness was called the damage load [10]. In the case of four-bolt and five-bolt specimens, which are illustrated by Figure 8c–f, bearing failures did not occur in the GFSs of [0/90] and [±45]. Since debonding failure occurred in the GFSs of [0/90] and [±45], it can be concluded that the bonding strength was smaller than the bearing strength in the four- or five-bolt specimens. A quantitative investigation to clarify bond strength will be conducted in the next study.

#### 5.2.2. Evaluation Strengthening Effect by Types of GFSs

**Figure 10:**
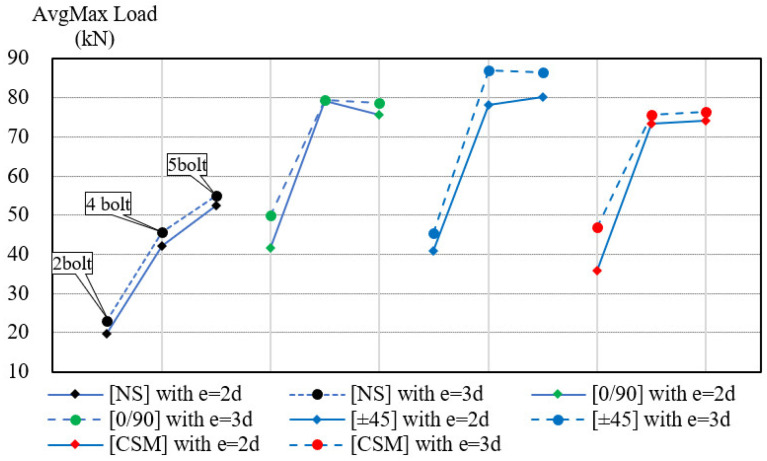
Average ultimate load of specimens.

Table 5 shows the obtained ultimate loads in the connection strength test. The average results of three samples for each designed specimen is illustrated by the line graphs in Figure 10. The maximum load of the GFSs was higher than the load in the NS specimens in all types of GFSs (the other parameters, the number of bolts and the end distance, were fixed). The effectiveness of the specimens after strengthening was also demonstrated by the [*P_st_/P_NS_*] ratio, which varied from 1.4 to 2.1. As shown in Table 6, the [CSM] effective ratio was lower than in any of other GFSs, at 40% with five--bolt specimens. The increasing ultimate load in the strengthening specimens proved the effectiveness of the solution for enhancing the serviceability of the PGFRP connection structure. Instead of increasing the volume of the material (length, width, or thickness), the use of GFS could be considered an advantageous method, especially with respect to the existing PGFRP structure.

#### 5.2.3. Evaluating the Strengthening Effect by Number of Bolts

There was a significant increase in connection strength when changing the bolt quantity from two bolts to four bolts. The effectiveness was also noticeable in NS in the case of changing four bolts to five bolts. However, the strengthening effect was trivial in GFS specimens when changing from four to five bolts. In the [0/90] and [±45] GFS types, the ultimate load in four-bolt connection specimens was higher than in five-bolt specimens because the area of bonding was decreased by one more bolt hole area. In [CSM] specimens, the tensile strength of GFS did not significantly change when adding one more bolt, from four bolts to five bolts. Due to the cross area of the failure section, the main factor causing net-tension failure, there was no change, and the ultimate load in [CSM] was not changed in these cases. On the other hand, the NS specimens obtained a failure mode change from MODE 1 (two and four bolts) to MODE 2 (five bolts, block shear). The length along the shear area was increased in the case of five bolts. Consequently, this made for better strength in comparison with two or four bolts specimens.

#### 5.2.4. Strengthening Effect Related to End Distance

In addition to the effect of the number of bolts and the type of GFS, the end distance *e* was also investigated in this study. Table 7 provides the percentages of increasing strength when changing from end distance *e = 2d* to *e = 3d.*

In the case of two-bolt specimens, all of the specimens were shown to have a high strengthening effect, with an increasing ratio ranging from 10.9% to 30.9%. The adding of end distance meant that the failure-out section of the CD layer was longer. Thus, the maximum load was stronger in *e = 3d* specimens.

In the four- or five-bolt specimens, only the [±45] specimens with four bolts showed an increase in connection strength (around a 12% increase).

In addition, the relative increasing values in the ultimate load trended lower in the four- or five-bolt specimens in comparison with the two-bolt specimens. This was because the absolute value of the ultimate load in the two-bolt specimens was much lower than that in the others. Therefore, it was more effective when increased by extending the end distance in the two-bolt specimens than in the four- or five-bolt specimens.

The bonding strength of the CD and the GFS layer was a major element when evaluation MODE 2 and MODE 5. These represented a failure mode that occurred in the four- or five-bolt specimens (except for the [CSM] specimens). The distribution and the area of effective bonding will be further investigated as a supplement to this study, for an increased understanding of this issue.

#### 5.2.5. Strengthening the Effect of Expanded GFS Areas

To investigate the effect of the bonding area, the GFS [±45] and [0/90] specimens were tested, as described in Section 4.2. The maximum loads in the connection testing are shown in Table 8.

The result of each the two types, [±45]_3-4 4d_ and [±45]_3-4 5d_, were compared with the results for corresponding specimens before being expanded, [±45]_3-4_. Similarly, [0/90]_3-4 4d_ and [0/90]_3-4 5d_ specimens were compared to [0/90]_3-4_ specimens, with values as provided in Table 5. Although the failure modes changed, the values of the maximum loads remained steady.

Based on the values of the ultimate loads and the failure modes, it can be concluded that the tensile strength and the bonding strength before expansion of the GFS area were approximately equal. The tensile strength depends only on the cross-section of the GFS, while the bonding strength ratio depends on the length of GFS in the specimens. Unlike the bonding strength, which is distributed in the whole GFM and the CD layer surface, the tensile strength is dependent on the minimum cross-section. Therefore, if the unloaded end was *3d*, the debonding failure gradually came first and net-tension did not occur. Then, when there was an increase in the length of the GFS in the unloaded end at *4d* or *5d*, the failure mode changed from debonding to net-tension in the [±45] specimens. This was because bond strength became higher than the tensile strength.

Among of failure modes, bearing failure is the safest for connections. This is because deformation develops gradually over a long period of loading increase. After reaching the ultimate load and when failure has occurred, the connection continues displacement but is not damaged immediately. The dimensions of the GFS can adjust to adapt to the design requirements. Increasing the thickness of the strengthening GFS sheet can prevent net-tension. Nevertheless, the debonding strength only depends on the properties of the PGFRP product. These criteria need to be calculated in the strengthening PGFRP connection.

This study has only explained the failure modes by reference to the maximum loads due to the complex working between the GFS and PGFRP components in the specimens. The bonding strength of PGFRPs will be quantitively investigated in future to completely demonstrate the tendency of the failure mode.

## 6. Conclusions

This study investigated the effectiveness of strengthening multi-bolted PGFRP connections by three kinds of GFSs. In the experiment, specimens were divided into groups according to the number of bolts, the end distances (e/d ratio), and the types of GFSs. Based on the results and the observed failure modes, there are some major conclusions, as follows:

- Five types of failure modes occurred in the 72 samples of the 24 types of specimens in the testing. In two- and four-bolt NS specimens, shear-out occurred in the whole cross-section. Block shear failure occurred at GFM and shear-out occurred at CD in the five-bolt NS specimens. The failure modes in the GFS specimens were all based on two-component failures mode. All the [CSM] specimens experienced net-tension failures in the GFS parts, while the failure modes in the [0/90] and [±45] specimens were dependent on the number of bolts. The combination between the bearing failures in GFS/GFM and the shear-out failures in the CD parts can be seen in the two-bolt specimens. On the other hand, the combination of shear-out failure in CD parts and debonding between CD and GFM parts was found in [0/90] and [±45] with four- and five-bolt specimens.
- The trend in failure modes that occurred in categories of specimens could be explained by separate measurements of component strength. The explanation of failure modes and the size of ultimate loads can be referred to in subsequent investigations of the design parameters of specimens and strengthening materials.
- The effectiveness of strengthening by GFSs was demonstrated by the results of the tests. The maximum loads in all the GFS specimens were higher than those of the NS specimens, ranging from 1.4 to 2.1 times higher. Therefore, the number of bolts in the NS specimens could be reduced by GFS strengthening (from four and five bolts to two bolts) in application. Furthermore, the end distance (connection area) in the NS specimens could be reduced by GFS-strengthening (from *e* = *3d* to *e* = *2d*).
- In comparison between types of GFS, the [0/90] specimens had the highest effect in the case of two bolts with both second and third end distances. Among the four- and five-bolt GFS specimens, the [±45] specimens had the highest effect; second were the [0/90] specimens. The types of [CSM] had the lowest effectiveness in all the GFS specimens. This result is necessary for consideration in the selection of GFS types in strengthening the PGFRP connection.
- The effectiveness of the increasing numbers of bolts was also investigated. There was an effectiveness in the NS specimens and the GFSs in cases of increasing from two to four bolts. However, this was an unremarkable result in regard to the GFS specimens with an increase from four to five bolts. This means that an increase in the number of bolts could be considered as a strengthening method for NS specimens.
- Increasing the end distance was shown to be an effective method for improvement in the case of two bolts for all NS and GFSs specimens.
- The failure mode is one of the safety factors for connections. Debonding failure depends on bond strength, which is a property of the PGFRP products. Therefore, it is necessary to investigate bond strength when designing the strengthening of bolted connections in PGFRPs.

The observed failure modes in the multi-bolt specimens were shown to be quite complicated, with five types of failure. It is necessary to conduct further investigation to analyze and sufficiently explain the failure tendency.

## Figures and Tables

**Table 1:** Minimum requirements for bolted-connection geometries.

Notation	Definition	Minimum Required Spacing (or Distance in Terms of Bolt Diameters)
*e*_min_	**End distance**	**Tension load**
	Single row of bolts	*4d*
	Two or three bolt rows	*2d*
	**End distance**	**Compression load**
	All connections	*2d*
*e*_2,min_	Edge distance	*1.5d*
*s*_min_	Pitch spacing	*4d*
*g*_min_	Gage spacing	*4d*
*g*_2,min_	Gage spacing with staggered bolts	*2d*

Where *d* is the nominal diameter of bolt. Minimum *e*_min_ may be reduced to *2d* when the connected member has a perpendicular element attached to the end that the connection force is acting towards.

**Table 2:** Test program for PGFRP connections.

Specimen	End Distance	No. of Bolts	*d_n_* (mm)	*t_UD_* (mm)	*t_GFM_* (mm)	*t*_GF_ (mm) avg	No. of Specimens	*d* (mm)
NS_2-2_	24	2	13.5	4	1	-	3	12
NS_2-4_	24	4	13.5	4	1	-	3	12
NS_2-5_	24	5	13.5	4	1	-	3	12
NS_3-2_	36	2	13.5	4	1	-	3	12
NS_3-4_	36	4	13.5	4	1	-	3	12
NS_3-5_	36	5	13.5	4	1	-	3	12
[0/90]_2-2_	24	2	13.5	4	1	1.259	3	12
[0/90]_2-4_	24	4	13.5	4	1	1.260	3	12
[0/90]_2-5_	24	5	13.5	4	1	1.260	3	12
[0/90]_3-2_	36	2	13.5	4	1	1.285	3	12
[0/90]_3-4_	36	4	13.5	4	1	1.246	3	12
[0/90]_3-5_	36	5	13.5	4	1	1.246	3	12
[CSM]_2-2_	24	2	13.5	4	1	1.650	3	12
[CSM]_2-4_	24	4	13.5	4	1	1.608	3	12
[CSM]_2-5_	24	5	13.5	4	1	1.570	3	12
[CSM]_3-2_	36	2	13.5	4	1	1.610	3	12
[CSM]_3-4_	36	4	13.5	4	1	1.590	3	12
[CSM]_3-5_	36	5	13.5	4	1	1.590	3	12
[±45]_2-2_	24	2	13.5	4	1	1.210	3	12
[±45]_2-4_	24	4	13.5	4	1	1.230	3	12
[±45]_2-5_	24	5	13.5	4	1	1.230	3	12
[±45]_3-2_	36	2	13.5	4	1	1.200	3	12
[±45]_3-4_	36	4	13.5	4	1	1.244	3	12
[±45]_3-5_	36	5	13.5	4	1	1.244	3	12

**Table 3:** Test program for PGFRP connections in GFS expansion.

Specimen	End Distance	No. of Bolts	*d_n_* (mm)	*t_UD_* (mm)	*t_GFM_* (mm)	*t*_GF_ (mm) avg	No. of Specimens	*d* (mm)
[±45]_3-4 4d_	36	4	14	4	1	1.244	3	12
[±45]_3-4 5d_	36	4	14	4	1	1.244	3	12
[0/90]_3-4 4d_	36	4	14	4	1	1.246	3	12
[0/90]_3-5 5d_	36	4	14	4	1	1.246	3	12

**Table 4:** Detail of failure mode in specimens.

Failure Mode	CD	GFM					GFS	
	Shear-Out	Net Tension	Block Shear	Shear-Out	Debonding	Bearing	Net Tension	Bearing
1	x			x				
2	x		x					
3	x				x			
4	x	x					x	
5	x					x		x

“x” indicates the type of failure mode that occurred in each component.

**Table 5:** The ultimate loads of PGFRP connections and the strengthening effects of GFSs (unit: kN).

Types	NS_2-2_	NS_2-4_	NS_2-5_	NS_3-2_	NS_3-4_	NS_3-5_
1	20.56	44.73	48.28	24.45	40.82	54.06
2	18.89	42.29	56.39	23.03	48.37	52.70
3	19.62	39.25	52.42	21.90	48.06	58.15
Avg	19.69	42.09	52.36	23.13	45.75	54.97
**Types**	**[0/90]_2-2_**	**[0/90]_2-4_**	**[0/90]_2-5_**	**[0/90]_3-2_**	**[0/90]_3-4_**	**[0/90]_3-5_**
1	44.70	72.33	78.10	51.78	79.66	83.42
2	38.19	84.82	71.68	49.21	75.41	76.98
3	42.14	80.64	76.96	48.48	83.46	75.32
Avg	41.68	79.26	75.58	49.82	79.51	78.57
Pst/PNS	2.12	1.88	1.44	2.15	1.74	1.43
**Types**	**[±45]_2-2_**	**[±45]_2-4_**	**[±45]_2-5_**	**[±45]_3-2_**	**[±45]_3-4_**	**[±45]_3-4_**
1	40.31	74.39	89.31	43.87	89.8	87.08
2	40.51	75.30	74.83	46.21	90.23	91.02
3	41.79	84.53	76.71	45.94	83.64	80.93
Avg	40.87	78.07	80.28	45.34	87.89	86.34
*Pst/P_NS_*	2.08	1.85	1.53	1.96	1.92	1.57
**Types**	**[CSM]_2-2_**	**[CSM]_2-4_**	**[CSM]_2-5_**	**[CSM]_3-2_**	**[CSM]_3-4_**	**[CSM]_3-5_**
1	36.97	70.18	72.92	45.50	79.06	74.82
2	32.58	72.57	77.04	47.01	77.88	78.29
3	38.25	77.08	72.07	48.6	70.08	76.04
Avg	35.93	73.27	74.01	47.04	75.67	76.38
*Pst/P_NS_*	1.83	1.74	1.41	2.03	1.65	1.39

*P_st_:* The ultimate loads of strengthened specimens. *P_NS_*: The ultimate loads of NS specimens.

**Table 6:** Strengthening effect of GFS.

No. Bolts	[±45]	[0/90]	[CSM]
*e = 2d* specimens			
2	108%	112%	83%
4	85%	88%	74%
5	53%	44%	41%
*e = 3d* specimens			
2	96%	115%	103%
4	92%	74%	65%
5	57%	43%	39%

**Table 7:** Comparison of the strengthening effect of *3d* end -distance specimens to *2d* end distance specimens.

No. Bolts	[±45]	[0/90]	[CSM]	NS
2	10.9%	19.5%	30.9%	17.5%
4	12.6%	0.3%	3.3%	8.7%
5	7.6%	4.0%	3.2%	5.0%

**Table 8:** Ultimate load of [0/90] and [±45] GFS expansion of the strengthening areas of specimens.

Types	[±45]_3-4 4d_	[±45]_3-4 5d_	[0/90]_3-4 4d_	[0/90]_3-4 5d_
1	87.72	79.988	75.368	77.372
2	91.44	87.416	75.36	68.316
3	86.72	80.712	69.496	75.04
Avg	88.62	82.71	73.41	73.58

## Data Availability

The data required to reproduce these findings cannot be shared at this time, as they also form part of an ongoing study.

## Appendix A

**Table A1:** Material properties.

Material Properties									
Fail. Mode	Specimens	Bearing Strg of GFM (Mpa)	Bearing Strg of GFS (Mpa)	Bearing Strength of (CD Mpa)	Shear-Out Strg of CD (Mpa)	Shear-Out Strg of (GFM)	Shear Strg of GFS (Mpa)	Tensile Strg of PGFRP (Mpa)	Tensile Strg of GFS (Mpa)
MODE 5	[0/90] 2 bolts 2d	199.71	146.03	260.49	11.68	81.91	86.00	536.00	420.00
	[0/90] 2bolts 3d	199.71	146.03	260.49	11.68	81.91	86.00	536.00	420.00
MODE 3	[0/90] 4 bolts 2d	199.71	146.03	260.49	11.68	81.91	86.00	536.00	420.00
	[0/90] 4 bolts 3d	199.71	146.03	260.49	11.68	81.91	86.00	536.00	420.00
	[0/90] 5 bolts 2d	199.71	146.03	260.49	11.68	81.91	86.00	536.00	420.00
	[0/90] 5 bolts 3d	199.71	146.03	260.49	11.68	81.91	86.00	536.00	420.00
MODE 5	[±45] 2 bolts 2d	199.71	154.56	260.49	11.68	81.91	94.92	536.00	169.00
	[±45] 2 bolts 3d	199.71	154.56	260.49	11.68	81.91	94.92	536.00	169.00
MODE 3	[±45] 4 bolts 2d	199.71	154.56	260.49	11.68	81.91	94.92	536.00	169.00
	[±45] 4 bolts 3d	199.71	154.56	260.49	11.68	81.91	94.92	536.00	169.00
	[±45] 5 bolts 2d	199.71	154.56	260.49	11.68	81.91	94.92	536.00	169.00
	[±45] 5 bolts 3d	199.71	154.56	260.49	11.68	81.91	94.92	536.00	169.00
MODE 4	[CSM] 2 bolts 2d	199.71	199.71	260.49	11.68	81.91	81.91	536.00	164.80
	[CSM] 2 bolts 3d	199.71	199.71	260.49	11.68	81.91	81.91	536.00	164.80
	[CSM] 4 bolts 2d	199.71	199.71	260.49	11.68	81.91	81.91	536.00	164.80
	[CSM] 4 bolts 3d	199.71	199.71	260.49	11.68	81.91	81.91	536.00	164.80
	[CSM] 5 bolts 2d	199.71	199.71	260.49	11.68	81.91	81.91	536.00	164.80
	[CSM] 5 bolts 3d	199.71	199.71	260.49	11.68	81.91	81.91	536.00	164.80
MODE 1	NS 2 bolt 2d	199.71		260.49	11.68	81.91		536.00	
	NS 2 bolt 3d	199.71		260.49	11.68	81.91		536.00	
	NS 4 bolt 2d	199.71		260.49	11.68	81.91		536.00	
	NS 4 bolt 3d	199.71		260.49	11.68	81.91		536.00	
MODE 2	NS 5 bolt 2d	199.71		260.49	11.68	81.91		536.00	
	NS 5 bolt 3d	199.71		260.49	11.68	81.91		536.00	

The component strength can be obtained by principal equation:

$$P_{i}=\tau_{i}A$$

where:

*τ_i_*: component strength in Table A1: properties of material that referred from Nhut [44,46] and the material testing
*A:* is the net area subject to each component strength:
  - Bearing strength: *A = dtn* with *d* and *n* are the diameter of bolt and number of bolts, *t* is the thickness of component layers.
  - Shear strength; tensile strength: *A = tL* with *t*; *T* is the thickness and total length of subject component layers

**Table A2:** Specimen’s parameter.

Specimens’ Parameters								
Fail. Mode	Specimens	Width *w* mm	End Distance *e* mm	Length of GFS *l* mm	No of Bolts *n* nos	Thk of CD *t_CD_* (mm)	Thk of GFS *t_GFS_* (mm)	Thk of GFM *t_GFM_* (mm)
MODE 5	[0/90] 2 bolts 2d	84.00	24.00	96.00	2.00	4.00	1.26	0.50
	[0/90] 2bolts 3d	84.00	36.00	120.00	2.00	4.00	1.29	0.50
MODE 3	[0/90] 4 bolts 2d	84.00	24.00	96.00	4.00	4.00	1.25	0.50
	[0/90] 4 bolts 3d	84.00	36.00	120.00	4.00	4.00	1.26	0.50
	[0/90] 5 bolts 2d	84.00	24.00	96.00	5.00	4.00	1.26	0.50
	[0/90] 5 bolts 3d	84.00	36.00	120.00	5.00	4.00	1.25	0.50
MODE 5	[±45] 2 bolts 2d	84.00	24.00	96.00	2.00	4.00	1.21	0.50
	[±45] 2 bolts 3d	84.00	36.00	120.00	2.00	4.00	1.20	0.50
MODE 3	[±45] 4 bolts 2d	84.00	24.00	96.00	4.00	4.00	1.23	0.50
	[±45] 4 bolts 3d	84.00	36.00	120.00	4.00	4.00	1.24	0.50
	[±45] 5 bolts 2d	84.00	24.00	96.00	5.00	4.00	1.23	0.50
	[±45] 5 bolts 3d	84.00	36.00	120.00	5.00	4.00	1.24	0.50
MODE 4	[CSM] 2 bolts 2d	84.00	24.00	96.00	2.00	4.00	1.65	0.50
	[CSM] 2 bolts 3d	84.00	36.00	120.00	2.00	4.00	1.61	0.50
	[CSM] 4 bolts 2d	84.00	24.00	96.00	4.00	4.00	1.61	0.50
	[CSM] 4 bolts 3d	84.00	36.00	120.00	4.00	4.00	1.59	0.50
	[CSM] 5 bolts 2d	84.00	24.00	96.00	5.00	4.00	1.57	0.50
	[CSM] 5 bolts 3d	84.00	36.00	120.00	5.00	4.00	1.59	0.50
MODE 1	NS 2 bolt 2d	84.00	24.00	96.00	2.00	4.00		0.50
	NS 2 bolt 3d	84.00	36.00	120.00	2.00	4.00		0.50
	NS 4 bolt 2d	84.00	24.00	96.00	4.00	4.00		0.50
	NS 4 bolt 3d	84.00	36.00	120.00	4.00	4.00		0.50
MODE 2	NS 5 bolt 2d	84.00	24.00	96.00	5.00	4.00		0.50
	NS 5 bolt 3d	84.00	36.00	120.00	5.00	4.00		0.50

**Table A3:** Component Strength.

Component Strength (kN)							
Fail. Mode	Specimens	*P_brCD_*	*P_brGFM/GFS_*	*P_soCD_*	*P_soGFS/GFM_*	*P_ntGFS/GFM_*	*P_ntPGFRP_*
MODE 5	[0/90] 2 bolts 2d	25.01	13.62	4.48	28.65	68.45	300.16
	[0/90] 2bolts 3d	25.01	13.80	6.72	43.62	69.68	300.16
MODE 3	[0/90] 4 bolts 2d	50.01	27.05	13.45	68.72	67.84	300.16
	[0/90] 4 bolts 3d	50.01	27.25	15.69	83.62	68.50	300.16
	[0/90] 5 bolts 2d	62.52	34.06	13.45	69.28	68.50	300.16
	[0/90] 5 bolts 3d	62.52	33.82	15.69	82.94	67.84	300.16
MODE 5	[±45] 2 bolts 2d	25.01	13.77	4.48	29.92	32.13	300.16
	[±45] 2 bolts 3d	25.01	13.70	6.72	44.60	31.94	300.16
MODE 3	[±45] 4 bolts 2d	50.01	27.84	13.45	73.18	32.51	300.16
	[±45] 4 bolts 3d	50.01	28.04	15.69	89.06	32.78	300.16
	[±45] 5 bolts 2d	62.52	34.80	13.45	73.18	32.51	300.16
	[±45] 5 bolts 3d	62.52	35.06	15.69	89.06	32.78	300.16
MODE 4	[CSM] 2 bolts 2d	25.01	20.61	4.48	33.81	39.68	300.16
	[CSM] 2 bolts 3d	25.01	20.23	6.72	49.78	38.95	300.16
	[CSM] 4 bolts 2d	50.01	40.41	13.45	80.12	38.91	300.16
	[CSM] 4 bolts 3d	50.01	40.07	15.69	95.87	38.58	300.16
	[CSM] 5 bolts 2d	62.52	49.61	13.45	78.67	38.21	300.16
	[CSM] 5 bolts 3d	62.52	50.09	15.69	95.87	38.58	300.16
MODE 1	NS 2 bolt 2d	25.01	4.79	4.48	7.86		300.16
	NS 2 bolt 3d	25.01	4.79	6.72	11.80		300.16
	NS 4 bolt 2d	50.01	9.59	13.45	19.00		300.16
	NS 4 bolt 3d	50.01	9.59	15.69	22.93		300.16
MODE 2	NS 5 bolt 2d	62.52	11.98	13.45	23.59		300.16
	NS 5 bolt 3d	62.52	11.98	15.69	27.52		300.16

ASCE [15] Block Shear Strength, *R_bs_*

When the connection force is concentric to the group of bolts, tensile and parallel to the direction of FRP material the nominal block shear strength for the multi-bolted connection shall be given by:

*R_bs_* = 0.5 (*A_ns_F_sh_* + *A_nt_* + *F^t^_L_*)

*φ_c_* = 0.45

where:

*F_sh_* = Characteristic in-plane shear strength of FRP material appropriate to the shear-out failure
*F^t^_L_* = Characteristic tensile strength of the FRP material in the longitudinal *A_ns_* = Net area subjected to shear
*A_nt_* = Net area subjected to tension, where the bolts are staggered the total deducted in c determining Ant shall be the greater of
  (a) the maximum of the sectional area in any cross-section perpendicular to the member axis, or
  (b) *t*(*nd_n_* − ∑*b_s_*)

where:

$$b_{s}\;\mathrm{is}\;\mathrm{the}\;\mathrm{lesser}\;\mathrm{of}\;r=\frac{s^{2}}{4g_{s}}\;\mathrm{or}0.65\;gs$$

*n* = Number of holes extending in any diagonal or zig-zag line progressively across the member or part of the member (*n**_ma_*_x_ = 3)
*d_n_* = Nominal diameter of hole

Calculated the value of block shear strength of NS five-bolt (NS_2-5_ and NS_3-5_) = 8.20 kN and 9.19 kN.
